# Toxicity of Methanolic Extracts of Seeds of *Moringa stenopetala*, *Moringaceae* in Rat Embryos and Fetuses

**DOI:** 10.1155/2021/5291083

**Published:** 2021-02-09

**Authors:** Daniel Teshome, Chalachew Tiruneh, Gete Berihun

**Affiliations:** ^1^Department of Anatomy, College of Medicine and Health Sciences, Wollo University, Dessie, Ethiopia; ^2^Department of Environmental Health, College of Medicine and Health Sciences, Wollo University, Dessie, Ethiopia

## Abstract

*Moringa stenopetala* is a medicinal plant that has been used in Ethiopian traditional medicine as a remedy for the treatment of hypertension, diabetes, and stomach pain. The study is aimed at assessing the toxicity of the methanol extracts of the seeds of *Moringa stenopetala* on the developing embryo and fetuses of rats. The seeds of *Moringa* were extracted by maceration using 80% methanol. The extract (250–1000 mg/kg) was orally administered to pregnant Swiss albino rats from days 6 to12 of gestation. Embryos and fetuses were recovered by laparotomy on gestational day 12 and day 20, respectively, and were assessed for developmental anomalies. On day 20, significant prenatal growth retardation such as reduced litter weight and crown-rump length were observed in near term fetuses of 1000 mg/kg treated rats. Litter weight in 1000 mg/kg and pair-fed control groups was 2.41 g ± 0.108 and 3.08 g ± 0.093, respectively. Delay in the development of an otic, optic, and olfactory system, as well as a reduction in a number of branchial bars, occurred on day 12 embryos of 1000 mg/kg treated rats. The rate of fetal resorption in 1000 mg/kg and pair-fed control groups was 1.6 ± 0.55 and 0.42 ± 0.52, respectively. There was also a high incidence of fetal death in the 1000 mg/kg treated group but it was not statistically significant. The offspring's of *Moringa*-treated rats did not show gross external malformations at all doses. These findings suggest that the methanol seed extract of *Moringa stenopetala* is not safe to rat embryos and fetuses. Its toxic effects were evidenced by a significant delay in embryonic and fetal development and an increase in fetal resorptions and fetal death.

## 1. Introduction

*Moringa stenopetala*, *Moringaceae* is a deciduous plant which is widely distributed throughout Southern Ethiopia. It is one of the medicinal plants widely used in popular culture for the treatment of various diseases including hypertension and diabetes mellitus. *M. stenopetala* belonged to the family *Moringaceae*, represented only by a single genus *Moringa.* This genus is represented by 14 species to which *M. Stenopetala* belongs [1, 2]. It is often referred to as the African *Moringa* tree because it is native only to Southern Ethiopia and Northern Kenya. However, it grows in many other parts of the tropics, and it is not as widely known as its close relative *Moringa oleifera*, but often considered as more desirable than *M. oleifera* [3].

Drought-resistant branched *Moringa* tree grows at an altitude range of 1000 to 1800 meters, where the leaves were eaten as a vegetable. It is widely distributed in Konso, Wolayta, Derashe, Gamogofa, Sidama, Bale, and Borana areas. This species is known by different vernacular names such as “Shiferaw” in Amharic, “Haleko” in Wollaytegna and Gamugna, “Shelagda” in Konso, and “Cabbage tree” in English [4].

The seeds of *M. stenopetala* contain crude fibers, ash, fatty acid, high crude proteins, neutral detergent fiber, and minerals such as iron, zinc, copper, and calcium in significant concentrations [5]. Phytochemical screening of the hot water infusion and aqueous crude extract of *M. stenopetala* revealed that it also contains high (+3) amount of alkaloids, flavonoids, tannins, and saponins [6].

Different parts of the plant were studied for nutritional and medicinal values. The flowers are good nectar sources for honey, the seeds are used in clearing muddy water, and the grinded wet or dried root was used to treat malaria [4].

The seeds of *M. stenopetala* contain edible oil that can be used for cooking and as salad dressings [7, 8]. Besides, *M. stenopetala* seeds have traditionally been used to purify turbid water in many tropical countries [9, 10] like its Asian counterpart *Moringa oleifera* [11]. It was reported that *M. stenopetala* seeds have better water purifying properties than *M. oleifera* seeds [10]. Crude extracts from the defatted seeds are reported to exhibit antimicrobial effects [12] and thus could be used to preserve different food and nonfood ingredients.

It has been reported that *M. stenopetala* has hypotensive [13], antihyperglycemic, and hypoglycemic effects [14–17]. It has also antileishmanial, antimicrobial, and antifertility activities [4, 18]. Additionally, *M. stenopetala* is important in the treatment of stomach pain and expulsion of retained placenta following birth [19].

The study conducted on alcoholic extract of *M. oleifera* stem bark exhibited significant antifertility activity (26.26 to 100%). It was found that the extract significantly reduced the number of live fetuses, whereas the resorption index and post implantation losses increased significantly. The percentage of abortion was found to be the highest (100%) with 100 mg/kg dose of alcoholic extract of *M. oleifera* stembark. In ovariectomized immature young rats, the extract showed significant estrogenic effect (vaginal opening, vaginal cornification, and increased uterine weight) and also prolonged the estrous cycle and particularly diestrous phase in the experimental animals at the dose of 100 mg/kg body weight of *M. oleifera* stem bark [20].

In another study, administration of *Moringa oleifera* extract orally in Charles Foster strain albino rats resulted in an abortifacient activity. In this study, *Moringa oleifera* was administered in an aqueous solution at a dose of 175 mg/kg body weight on days 5-10 of gestation. This study reported 100% abortifacient activity of *Moringa oleifera.* The maternal body weight gain was significantly reduced in the treatment group compared to the control group. There was also a high prevalence of fetal resorptions in *Moringa*-treated groups [21].

The beneficial use of *M. stenopetala* has been extensively studied and reported. However, there has not been any report on the developmental toxicity of *M. stenopetala* seeds yet. Therefore, the present study is aimed at investigating the possible toxicity of *M. stenopetala* seeds in rat embryos and fetuses. Swiss albino rat was used as an animal model because the rat has become a standardized physiological and toxicological model, particularly in pharmaceutical industries. The finding of this study will help in the formulation of regulatory legislation regarding the use of this plant. The outcome of the study will also serve as a premise for further investigation on this plant and may help to develop intervention strategies to control the use of *M. stenopetala* by pregnant mothers.

## 2. Materials and Methods

### 2.1. Plant Materials

Fresh and uncrushed seeds of *M. stenopetala* were collected from Arbaminch about 505 km South of Addis Ababa, Ethiopia, on March 2018. The plant material was authenticated by a taxonomist in the EPHI, and a voucher number AL-001 was deposited in the herbarium for future reference.

### 2.2. Plant Material Preparation and Extraction

The dried seeds of *M. stenopetala* were finely pulverized into powder and then macerated in 80% methanol for 48 hours. The solution was filtered, and methanol was evaporated using rotary evaporator (Büchi Rota Vapor R-205, Switzerland) under reduced pressure, and the concentrated extract was dried using lyophilizer (Operan Lyophilizer, Korea) to completely remove the solvent residue. The average yield of dried *M. stenopetala* seed extract from the dried seed was 15.95%. The extract was kept in a tightly sealed container at -20°C until use.

### 2.3. Experimental Animals

Throughout the course of this investigation, healthy nulliparous 50 female and 25 male Swiss albino rats (weighing 200 to 220 g and age of 12-14 weeks) were used. The rats were obtained from the laboratory animal breeding facility of the EPHI.

The animals were housed in suspended stainless steel cages in an environmentally controlled room (22-23°C) and relative humidity of 50% ± 10. A cycle of 12 hr of light and 12 hr of dark was maintained at all times. They were acclimatized for 5 days. During the period of adaptation, all the animals were provided with free access to standard pellet laboratory diet and water *ad libitum.* The handling of animals and all experimental procedures were carried out according to internationally accepted guidelines (OECD, 2008) [22].

For mating purpose, the female Swiss albino rat should be added into the male cage in 1 to 2 ratios. After an overnight mating, a female rat was inspected for the presence of a copulatory plug the following morning and vaginal smears were taken for microscopic determination of the presence of sperm. The presence of spermatozoa in the vaginal smear was considered as day 0 of gestation [23].

### 2.4. Acute Toxicity Study

The extracts of *M. stenopetala* seeds were evaluated for possible toxic effect in female Swiss albino rats at a dose of 5000 mg/kg body weight according to OECD guidelines No. 425 [22]. On the basis of oral reports of high consumption of the leaves and seeds for diet mixed with cultural foods, the chosen dose of extract was highest (5000 mg/kg body weight). On the first day of the test, one female Swiss albino rat fasted for 3 hours was given 5000 mg/kg of the crude extract orally using oral gavages. Then, the rat was kept under strict observation for physical or behavioral changes for 24 hr, with special attention during the first 4 hours. Because mortality was not observed in the first rat, other two female rats fasted for 3-4 hours were sequentially given a single dose of 5000 mg/kg of the seed extract and then observed in the same manner. The observation was continued for a total of 14 days for any sign of toxicity and mortality.

### 2.5. Grouping and Dosing of Pregnant Rats

The pregnant rats were randomly divided into five groups (3 experimental and 2 control groups) with each group consisting of five rats. Group I was treated with distilled water and served as the pair-fed control group. Groups II, III, and IV were treated with 250 mg/kg, 500 mg/kg, and 1000 mg/kg of the crude *M. stenopetala* seed extracts, respectively. Group V was unrestricted-fed *ad libitum* group.

The various doses of the *M. stenopetala* extract were selected based on the result of acute toxicity study. The extract was weighed and mixed with distilled water and continuously shacked with a vortex shaker. Final volume was 2 ml/100 g with the vehicle (distilled water), and oral gavage was used for oral administration [23]. The treatment period was from days 6 to 12 of gestation. The rationale for administration of the extract from day 6 through day 12 of gestation is because this period represents a period of active embryogenesis and organogenesis [24].

Each animal in the respective experimental group (groups II, III, and IV) received the control diet of equal amount. The pair-fed control group had the same diet in the same amount as the experimental groups and kept in the same environment except the *M. stenopetala* extracts, which were given only to the experimental groups (groups II, III, and IV). The daily food intake of each animal was recorded every morning, and animals' weight was recorded either on days 0, 6, and 12 of gestation (for day 12 experiment) or on days 0, 6, 12, and 20 of gestation (for day 20 experiment) [23].

### 2.6. Day 12 *Moringa stenopetala* Seed Extract Experiment

This experiment was designed to assess the possible developmental toxicity of *M. stenopetala* seeds in 12-day-old whole rat embryos. The experiment was expected to show any growth and developmental anomalies that might not have been apparent in the near-term fetuses due to possible compensatory growth and development.

On day 12 of gestation, at 12 : 00 hours, the pregnant rats were euthanized by inhalation of 4% diethyl ether. This was achieved by putting the animal in a tight desiccator jar having cotton rinsed with high dose of diethyl ether. The rats were kept in the desiccators until they lose their consciousness. In addition, cervical dislocations were done to lose their consciousness. Once the rats lose their consciousness, they were removed from the desiccators and placed in a supine position on an operating board. The limbs were stretched and fixed; the abdominal cavity was opened by abdominal midsagittal skin incision. The flap of the skin and the abdominal muscles on both sides were reflected laterally and held with pins. Then, the uterine horns were removed and placed in Hank's balanced salt solution. The pregnancy outcomes such as the number of implantation and resorption sites were counted. The uterine horns were then incised along the antimesometrial border to reveal the embryos. With the aid of fine forceps and a dissecting microscope, the membranes surrounding the embryos were removed to reveal the underlying visceral yolk sac. The yolk sac circulation and development were evaluated. The embryos were then explanted, and embryonic growth such as development of the circulatory, nervous, visual, auditory, olfactory, and skeletal systems, as well as craniofacial development, was assessed quantitatively on the basis of 16 recognizable developmental endpoints (morphological scores), according to the criteria of Brown and Fabro [25]. In addition, the numbers of somites were also counted.

### 2.7. Day 20 *Moringa stenopetala* Seed Extract Experiment

This experiment was designed to establish the possible developmental toxicity of *M. stenopetala* during gestation in 20-day-old rat fetuses.

On a gestational day 20, gravid females were euthanized by inhalation of 4% diethyl ether. The abdominal cavity was opened by abdominal midsagittal skin incision. The flap of the skin and the abdominal muscles on both sides were reflected laterally and held with pins. Then, the uterine horns were exposed and examined intact. The pregnancy outcomes such as the number of implantation sites were determined by counting the metrial glands, which are yellowish nodules, located along the mesometrial margin of the uterine horns. The metrial nodules, which were not occupied by living or recently dead fetuses, were representing the number of prior resorptions. The numbers of live or dead fetuses were determined by applying gentle pressure on them. The uterine horns were incised along the antimesometrial border to reveal the fetuses, fetal membranes, and the placenta. Fetuses were then recovered and dissected free of the placenta. The crown-rump length (CRL) was measured. Following these measurements, the fetuses were fixed in Bouin's solution (aqueous saturated solution of picric acid 75%, formalin 25%, and glacial acetic acid 5%) for gross external examination [23].

### 2.8. External Evaluation

Bouin's fixed fetuses were examined head to tail for gross external malformations under a dissecting microscope. The parameters assessed were as follows:

- Craniofacial development (exencephaly, anencephaly, microphthalmia, and anophthalmia)
- Development of the limbs (syndactyly, adactyly, and polydactyly)
- Vertebral column (neural tube defect, kyphosis, and scoliosis)
- Tail development (missing tail)
- External genitalia

### 2.9. Statistical Analysis

The data were expressed as mean ± standard error of the mean (SEM). Between and within group analyses were carried out using one-way ANOVA followed by Tukey's post hoc multiple comparison test. The results were considered to be significant when the *P* value was less than 0.05. The SPSS version 23 software was used for data processing and analysis.

## 3. Results

### 3.1. Acute Oral Toxicity Study

The acute toxicity study of *Moringa stenopetala* seed extract did not show mortality in the animals at a dose of 5000 mg/kg during the observation period. Thus, the median lethal dose (LD50) of the seed extract is greater than 5000 mg/kg. Besides, the toxicity study of *Moringa stenopetala* seed extract did not reveal any signs of toxicity: behavioral, neurological, autonomic, or physical changes. Here, we can select doses by dividing our LD50, i.e., 5000 mg/kg with 5, 10, and 20 resultants. So, the doses were 1000, 500, and 250 mg/kg/bw of extract.

### 3.2. Day 12 *Moringa* Experiment

#### 3.2.1. Maternal Food Intake and Weight Gain

Maternal daily food intake was slightly reduced during the treatment period (days 6-12) at the dose of 500 mg/kg and 1000 mg/kg compared to pair-fed control groups. But, there were no significant differences in daily food intake between the treatment and pair-fed control groups. The daily food intake in the *ad libitum* control group was significantly higher than any any of the treatment groups. Despite similar daily food intakes, treatment of pregnant rats with *Moringa* seed extracts at a dose of 250 mg/kg/day, 500 mg/kg/day, and 1000 mg/kg/day from days 6 to 12 of gestation shows a dose-dependent reduction in maternal weight gain. The maternal weight gain was significantly lower in 1000 mg/kg *Moringa*-treated group compared to all the other groups (Table 1).

#### 3.2.2. Pregnancy Outcomes and Embryonic Growth

The data regarding pregnancy outcome were analyzed using one-way ANOVA. Five Swiss albino rats within each group were analyzed. The result showed that the number of implantation site did not appear to be different from all the other groups. There was high incidence of fetal resorptions at a dose of 1000 mg/kg compared to all the other groups.

Embryonic growth indices used were the number of somites and morphological score. The number of somites present was considered as one of the most important criteria for assessing embryonic growth. Compared to all other groups, the number of somites following treatment with 1000 mg/kg *Moringa* seed extract was significantly decreased (*P* < 0.05). Embryos of 1000 mg/kg *Moringa* leaf extract-treated group show a significant reduction in the morphological score compared to all the other groups (Table 2).

#### 3.2.3. Embryonic Development

Developmental status of the primordia of the various systems was assessed according to the morphological scoring system of Brown and Fabro. The results are summarized in Table 3. In rats treated with 1000 mg/kg/day of *Moringa* leaf extracts, the yolk sac of their embryos was not obliterated and vitelline artery and veins were not separated. There was a full yolk sac plexus of vessels compared to *ad libitum* group. An embryo of M1000 mg/kg-treated group shows a significant decrease in the degree of flexion when compared with pair-fed control, M250 mg/kg, and *ad libitum* groups.

There were no significant differences in the development of cardiac primordium between the groups. The posterior neuropore was closed in all of the treatment and control groups. With respect to the closure of anterior neuropore, there were no significant differences between any of the groups. All embryos revealed a completely fused mesencephalon. There was no significant difference among the groups. A visible telencephalic evagination was present in all embryos. In *ad libitum* group, there was a well-elevated telencephalic hemisphere. But, there was no significant difference between all of the groups.

In 500 mg/kg/day and 1000 mg/kg/day *Moringa* seed-treated groups, there was a significant delay in the development of otic, optic, and olfactory systems compared to pair-fed control and *ad libitum* groups. A significant decrease in the number of branchial bars was observed in M500 mg/kg and M1000 mg/kg treated groups as compared with pair-fed control and *ad libitum* groups. There was also a slight decrease in the number of branchial bars in M250 mg/kg treated group but not statistically significant.

The maxillary process was demarcated and visible cleft anterior to the branchial bar I in all of the embryos. No significant differences were observed between the groups. In M1000 mg/kg/day treated group, there was no sign of mandibular development from the bar I compared to all other groups. With respect to forelimb and hind limb development, no significant differences were observed between the groups (Table 3).

### 3.3. Day 20 *Moringa* Experiment

#### 3.3.1. Maternal Food Intake and Weight Gain

During the pretreatment period (days 1-5), there were no significant differences in maternal food intake between treatment and control groups. During treatment period (days 6-12) and posttreatment period (days 13-20), there was a dose-dependent reduction in daily food intake compared to both pair-fed control and *ad libitum* groups. But, it was not statistically significant.

During the treatment period, the maternal weight gain in the M1000 mg/kg treated group was significantly lower (*P* < 0.05) compared to both pair-fed control and *ad libitum* groups. Significant reductions in maternal weight gains were also seen in M500 mg/kg and M1000 mg/kg treated groups during days 6-20 compared to both pair-fed control and *ad libitum* groups (Table 4).

#### 3.3.2. Pregnancy Outcomes

The numbers of fetuses in M250 mg/kg, M500 mg/kg, and M1000 mg/kg *Moringa*-treated groups were 43, 43, and 41, respectively. There was a dose-dependent reduction in the number of fetuses and implantation sites among the groups but not statistically significant. With respect to fetal resorptions, there was a high incidence of fetal resorptions in M1000 mg/kg treated groups compared to all the other groups. The number of live fetuses was significantly decreased in M1000 mg/kg treated groups compared to both pair-fed control and *ad libitum* groups. Compared to all other groups, there was also a high incidence of fetal death in this group (M1000 mg/kg). But, the relationship was not statistically significant at the level of 0.05 (ANOVA) (Table 5).

#### 3.3.3. Fetal Growth

The growth of fetus at term was significantly (*P* < 0.05) affected at the highest doses of treatment groups compared to the pair-fed control and the *ad libitum* group. The CRL was significantly lower in M500 mg/kg and M1000 mg/kg treated groups compared to the pair-fed control and the unrestricted *ad libitum* group (Table 6). Gross external developmental anomalies were not seen on craniofacial development, development of the limbs, and vertebral column, like neural tube defect, tail development (missing tail), and external genitalia.

## 4. Discussion

In the present study, the developmental toxicity of *M. stenopetala* methanol seed extracts in rat embryo and fetus was investigated.

Treatment with various doses of the extract was well tolerated by all animals, as there were no toxic effects observed by gross visual observation of the animals throughout the experiment. There was no death and apparent behavioral changes recorded during the course of the experiment in all treatment groups as compared to the control groups. But, treatment of pregnant rats at the highest dose (1000 mg/kg/day) showed a significant reduction in maternal weight gain compared to both pair-fed control and *ad libitum* groups. This finding is in line with the reports of previous studies conducted on toxicological evaluations of the crude extracts and fractions of *M*. *stenopetala* leaves [2]. This study also revealed a significant reduction in maternal weight gain at the same doses of *M. stenopetala* (1000 mg/kg was used) [2]. Our study finding is also in agreement with another investigation on subacute toxicity of crude extracts of *M. oleifera* in rats [26]. However, our findings were contrary to reports of a study conducted on the chronic effects of *M. stenopetala* on blood parameters and histopathology of liver and kidney in mice [27]. This discrepancy may be due to variation in animal model and duration of administration of the extract.

In this study, the number of implantation sites at the highest dose was not statistically significant compared with all groups. However, compared with the *ad libitum* group, the M1000 mg/kg treatment group had a higher incidence of fetal absorption. This finding is consistent with the data reported on the abortion activity of *Moringa oleifera* in rats [21]. The fact that the active compounds of the plants in the two plants are the same can explain this effect.

The growth of embryos was evaluated by counting the number of somites and morphological score. In the current study, the number of somites and morphological score at the highest dose (1000 mg/kg/day) was significantly reduced compared with all other groups. Embryo development was also evaluated according to the morphological scoring system of Brown and Fabro to check the expected development of different systems at the end of organogenesis [25]. The results showed that compared with the *ad libitum* control group and the pair-fed control group, a statistically significant delay in embryo development was observed at a dose of 1000 mg/kg/day of Moringa seed. At this dose (1000 mg/kg), delays in the development of the yolk sac, ear system, optic nervous system, and olfactory system were also observed. Concerning craniofacial development, compared with the pair-fed control group and the *ad libitum* control group, the degree of flexion and the number of branchial bars in the M1000 mg/kg/day treatment group were significantly reduced. Also, delay of mandibular process development in the M1000 mg/kg/day treatment group was observed. This may be due to the presence of alkaloids in *Moringa stenopetala* seed extract, which may cause delays in development [28]. However, there were no differences in the development of the primordia of the heart, forelimbs, and hind limbs and the development of the central nervous system including the tail neural tube, forebrain, midbrain, and hindbrain among the groups.

Compared with the pair-fed and ad libitum control groups, prenatal growth delays, such as reduced litter weight and crown-rump length, were observed in recent fetuses of animals treated with high-dose (1000 mg/kg/day) *Moringa* seed extract. In addition, frequencies of fetal resorptions per litter and the number of dead fetuses also increased statistically significantly. This study also revealed a dose-dependent decrease in the number of fetuses in each dam, but this was not statistically significant. This developmental delay in prenatal growth may be due the presence of high amount of alkaloid in *Moringa stenopetala* seed which may cause developmental defects through the disruption of cholinergic neurotransmission [28]. However, in all fetuses of animals treated with all doses of *Moringa stenopetala*, no developmental anomalies and general external organ abnormalities were observed.

## 5. Conclusion

Findings in the developmental toxicity test suggest that the methanolic seed extracts of *M. stenopetala* are not safe to rat embryos and fetuses at the highest dose. Its toxic effects were evidenced by significant delay in embryonic and fetal development and increase in fetal resorptions and fetal death. Therefore, excessive intake of *M. stenopetala* seed may be unsafe.

## Figures and Tables

**Table 1:** Mean daily food intake and maternal weight gain following treatment of pregnant rats with *Moringa* seed extracts in the day 12 experiment.

Groups	Daily food intake (g/day)		Maternal weight gain per dams (g)
	Days 1-5	Days 6-12	
G-I	15.63	16.02	5.89 ± 0.34
G-II	15.68	15.82	5.53 ± 0.71
G-III	15.75	15.72	5.16 ± 1.18
G-IV	15.71	15.68	4.51 ± 0.51^a^
G-V	15.77	17.01^b^	7.65 ± 1.29

Results are summarized as mean ± SDM. ^a^Significantly different (*P* < 0.05) from *ad libitum*, pair-fed control, M250 mg/kg, and M500 mg/kg groups. ^b^Significantly different (*p* < 0.05) from M250 mg/kg, M500 mg/kg, and M100 mg/kg groups.

**Table 2:** Pregnancy outcome and embryonic growth following treatment of pregnant rats with *Moringa* seed extracts in the day 12 experiment.

Groups	Pregnancy outcomes		Embryonic growth	
	Implantation sites per litter	Resorptions per litter	Number of somites	Morphological scores
G-I (pair-fed)	10 ± 1.54	0.32 ± 0.54	30.41 ± 1.83	44.24 ± 2.1
G-II (M250)	9.7 ± 1.4	0.38 ± 0.51	30.63 ± 1.68	44.05 ± 2.12
G-III (M500)	9.68 ± 1.3	0.36 ± 0.45	30.82 ± 1.49	43.81 ± 2.36
G-IV (M1000)	9.5 ± 1.42	1.38 ± 0.63^a^	27.01 ± 1.38^a^	41.04 ± 2.43^a^
G-V (*ad libitum*)	9.62 ± 1.31	0.28 ± 0.45	30.67 ± 1.23	44.87 ± 2.22
*F*-statistic	1.241	4.071	51.81	11.62
*P* value	0.33	0.014	<0.001	<0.001

Results are summarized as mean ± SDM. ^a^Significantly different (*P* < 0.05) from *ad libitum* control, pair-fed control, M250 mg/kg, and M500 mg/kg groups.

**Table 3:** *In vivo* development of rat embryo following treatment with *Moringa* seed extracts in the day 12 experiment.

Morphological end point	Groups				
	G-I	G-II	G-III	G-IV	G-V
No. of fetus/group	47	44	41	39	46
Yolk sac	3.57 ± 0.5	3.59 ± 0.5	3.63 ± 0.49	3.31 ± 0.47^a^	3.54 ± 0.5
Flexion	2.7 ± 0.46	2.68 ± 0.5	2.59 ± 0.49	2.31 ± 0.5^a,b,c^	2.7 ± 0.465
Heart	3.55 ± 0.5	3.55 ± 0.5	3.44 ± 0.502	3.44 ± 0.502	3.57 ± 0.5
CND	4 ± 0.00	4 ± 0.00	4 ± 0.00	4 ± 0.00	4 ± 0.00
Hind brain	3.45 ± 0.5	3.55 ± 0.5	3.37 ± 0.49	3.44 ± 0.5	3.43 ± 0.5
Mid brain	3.49 ± 0.5	3.52 ± 0.5	3.54 ± 0.51	3.33 ± 0.48	3.33 ± 0.48
Fore brain	3.68 ± 0.5	3.66 ± 0.5	3.68 ± 0.47	3.72 ± 0.46	3.74 ± 0.44
Otic system	3.57 ± 0.5	3.49 ± 0.5	3.2 ± 0.53^a,b^	3.15 ± 0.48^a,b^	3.59 ± 0.49
Optic sys	2.62 ± 0.5	2.41 ± 0.5	2.07 ± 0.8^a,b^	1.8 ± 0.94^a,b^	2.59 ± 0.5
Olfactory system	0.66 ± 0.5	0.52 ± 0.5	0.41 ± 0.5^a,b^	0.33 ± 0.5^a,b^	0.67 ± 0.4
Branchial bars	3.43 ± 0.5	3.39 ± 0.6	3.02 ± 0.6^a,b^	2.77 ± 0.5^a,b^	3.46 ± 0.55
Maxillary process	1.38 ± 0.5	1.48 ± 0.5	1.41 ± 0.5	1.33 ± 0.48	1.46 ± 0.5
Mandibular process	0.55 ± 0.5	0.58 ± 0.5	0.46 ± 0.5	0.00 ± 0.00^a,b,c,d^	0.54 ± 0.5
Forelimb	2 ± 0.00	2 ± 0.00	2 ± 0.00	2 ± 0.00	2 ± 0.00
Hind limb	2 ± 0.00	2 ± 0.00	2 ± 0.00	2 ± 0.00	2 ± 0.00

Statistical differences between the groups were analyzed by Duncan's multiple range tests. Results are expressed as mean ± SDM. ^a^*P* < 0.05 compared to pair-fed control. ^b^*P* < 0.05 compared to the *ad libitum*. ^c^*P* < 0.05 compared to M250 mg/kg. ^d^*P* < 0.05 compared to M500 mg/kg. CND: caudal neural tube.

**Table 4:** Daily food intakes and maternal weight gains of animals in day 20 *Moringa* seed extract experiment.

Groups	Daily food intake (g/day)			Maternal weight gain (g/day)	
	Days 1-5	Days 6-12	Days 13-20	Days 6-12	Days 6-20
G-I	15.19 ± 0.17	15.78 ± 0.32	16.67 ± 0.35	5.78 ± 0.76	15.87 ± 0.81
G-II	15.18 ± 0.18	15.46 ± 0.12	16.22 ± 0.21	6.47 ± 0.67	16.06 ± 0.76
G-III	15.17 ± 0.15	15.34 ± 0.15	16.13 ± 0.08	5.83 ± 0.83	14.15 ± 0.56
G-IV	15.37 ± 0.07	15.33 ± 0.03	16.03 ± 0.04	4.41 ± 0.79^a^	10.73 ± 0.57^a^
G-V	15.31 ± 0.08	15.79 ± 0.15	16.75 ± 0.42	6.89 ± 0.67	18.48 ± 0.85
*F*-statistic	2.16	8.44	78.72	12.26	84.98
*P* value	0.111	0.072	0.061	<0.001	<0.001

Results are expressed as mean ± SDM. ^a^Results are significantly different (*P* < 0.05) from both *ad libitum* and pair fed*-*control groups (ANOVA).

**Table 5:** Pregnancy outcomes of day 20 *Moringa* seed extract experiment.

Groups	No. of fetuses	Implantation sites	Number of resorptions/litter	Number of live fetus/dam	Number of dead fetus/dam
G-I	49	9.2 ± 0.84	0.42 ± 0.52	9.1 ± 0.89	0.4 ± 0.55
G-II	43	8.6 ± 0.89	0.45 ± 0.51	8.4 ± 0.55	0.33 ± 0.45
G-III	43	8.8 ± 0.84	0.44 ± 0.51	8.4 ± 0.55	0.4 ± 0.55
G-IV	41	8 ± 1.22	1.6 ± 0.55^a^	7.6 ± 0.89^b^	0.7 ± 0.447
G-V	48	9.4 ± 1.14	0.42 ± 0.52	9 ± 0.71	0.4 ± 0.55
*F*-statistic	0.841	2.46	4.8	4.333	0.923
*P* value	0.501	0.079	0.007	0.011	0.47

Results are summarized as mean ± SDM. ^a^Significantly different (*P* < 0.05) from *ad libitum*, pair-fed control groups, M250 mg/kg, M500 mg/kg, and M1000 mg/kg groups (ANOVA). ^b^Significantly different (*P* < 0.05) from *ad libitum* and pair-fed control groups.

**Table 6:** Mean fetal growth following treatment with *Moringa* seed extracts in the day 20 experiment.

Groups	Fetal growth	
	Litter weight/fetus (g)	CRL/fetus (cm)
G-I	3.11 ± 0.078	3.08 ± 0.122
G-II	3.04 ± 0.093	3.03 ± 0.141
G-III	2.95 ± 0.117^a^	2.98 ± 0.125^a^
G-IV	2.41 ± 0.108^a^	2.81 ± 0.167^a^
G-V	3.08 ± 0.093	3.08 ± 0.104
*F*-statistic	367.4	30.9
*P* value	<0.001	<0.001

Results are summarized as mean ± SDM. ^a^Significantly different (*P* < 0.05) from *ad libitum* and pair-fed control groups (ANOVA).

## Data Availability

The datasets used and/or analyzed during the current study are available from the corresponding author on reasonable request.

## Abbreviations

AAU: Addis Ababa University
ANOVA: Analysis of variance
CRL: Crown-rump length
EPHI: Ethiopian Public Health Institute
*M.*: *Moringa*
OECD: Organization of Economic Cooperation and Development
SDM: Standard deviation of mean
TMMRD: Traditional and Modern Medicine Research Directorate.
